# Predictors of SARS-CoV-2 IgG Spike Antibody Responses on Admission and Clinical Outcomes of COVID-19 Disease in Fully Vaccinated Inpatients: The CoVax Study

**DOI:** 10.3390/jpm12040640

**Published:** 2022-04-15

**Authors:** Eleni Livanou, Erasmia Rouka, Sotirios Sinis, Ilias Dimeas, Ioannis Pantazopoulos, Dimitrios Papagiannis, Foteini Malli, Ourania Kotsiou, Konstantinos I. Gourgoulianis

**Affiliations:** 1 Department of Respiratory Medicine, Faculty of Medicine, University of Thessaly, 41110 Larissa, Greece; elivanou97@gmail.com (E.L.); sinis1995@windowslive.com (S.S.); dimel13@hotmail.com (I.D.); pantazopoulosioannis@yahoo.com (I.P.); kgourg@med.uth.gr (K.I.G.); 2 Faculty of Nursing, University of Thessaly, 41500 Larissa, Greece; dpapajon@gmail.com (D.P.); mallifoteini@yahoo.gr (F.M.); raniakotsiou@gmail.com (O.K.)

**Keywords:** breakthrough COVID-19 hospitalizations, clinical outcomes, SARS-CoV-2 IgG Spike responses, vaccine-induced immunity

## Abstract

Background: SARS-CoV-2 vaccines have shown high efficacy in protecting against COVID-19, although the determinants of vaccine effectiveness and breakthrough rates are yet to be determined. We aimed at investigating several factors affecting the SARS-CoV-2 IgG Spike (S) antibody responses on admission and clinical outcomes of COVID-19 disease in fully vaccinated, hospitalized patients. Methods: 102 subjects were enrolled in the study. Blood serum samples were collected from each patient upon admission for the semiquantitative determination of the SARS-CoV-2 IgG S levels with lateral flow assays. Factors influencing vaccine responses were documented. Results: 27 subjects had a negative antibody test upon hospital admission. Out of the 102 patients admitted to the hospital, 88 were discharged and 14 died. Both the absence of anti-S SARS-CoV-2 antibodies and poor clinical outcomes of COVID-19 disease were associated with older age, lower Ct values, and a shorter period between symptom onset and hospital admission. Ct values and time between symptom onset and hospitalization were independently associated with SARS-CoV-2 IgG S responses upon admission. The PaO2/FiO2 ratio was identified as an independent predictor of in-hospital mortality. Conclusions: Host- and disease-associated factors can predict SARS-CoV-2 IgG S responses and mortality in hospitalized patients with breakthrough SARS-CoV-2 Infection.

## 1. Introduction

The ongoing COVID-19 pandemic, caused by the severe acute respiratory syndrome coronavirus 2 (SARS-CoV-2), has become a significant global public health issue [1]. As of 28 January 2022, there has been 364,191,494 confirmed cases of COVID-19 worldwide, with 5,631,457 deaths reported to the World Health Organization (https://covid19.who.int/, accessed: 29 January 2022). SARS-CoV-2 causes a variety of symptoms ranging from mild, flu-like symptoms to severe pulmonary damage with respiratory distress syndrome and death [2]. Subjects with pre-existing comorbidities including obesity, cardiovascular disease, type 2 diabetes mellitus (T2D), and chronic renal and lung disease are at an increased risk of developing acute respiratory distress syndrome (ARDS), requiring mechanical ventilation and admission to the intensive care unit (ICU) [3].

Vaccination is the most cost-effective medical intervention, preventing millions of deaths every year [4]. Vaccines have greatly reduced the burden of infectious diseases [5] and constitute an important tool for limiting epidemics caused by emerging pathogens [4]. Vaccine-induced immunity is mediated by the complex interaction of innate, humoral, and cell-mediated immunity [6]. Vaccines operate by inducing an immune response and, as a result, an immunological memory, which protects against infection or disease [7].

The approved SARS-CoV-2 vaccines have been highly efficient in protecting against COVID-19 [8,9], although the determinants of vaccine effectiveness and breakthrough rates are yet to be determined, especially in light of the emergence of viral variants of concern [10]. Antibody responses to SARS-CoV-2 vaccines have been shown to be affected by a variety of factors, including age [11], sex [12], central obesity [13], hypertension [11,13], cancer [14], dyslipidemia [13], and smoking habits [11,13].

However, there is a plethora of factors that influence humoral and cellular vaccine responses in humans. These include intrinsic host factors as well as extrinsic, environmental, behavioral, nutritional, and vaccine factors [6]. Variables affecting the immune response to the SARS-CoV-2 vaccination have not been extensively investigated. In this study, we examine several factors that may have an impact on SARS-CoV-2 IgG Spike (S) antibody responses and the outcome of COVID-19 disease in fully vaccinated, hospitalized patients.

## 2. Materials and Methods

### 2.1. Study Design

Within two months we prospectively studied 102 fully vaccinated adult patients (71 men, 31 women) who were admitted to the COVID-19 Department of the University Hospital of Larissa, Greece. SARS-CoV-2 infection was verified by real-time reverse-transcription polymerase chain reaction (RT-PCR). Several factors that influence vaccine responses were documented [6] (Table 1). The patients were monitored until hospital discharge or death. The study was approved by the Institutional Research Ethics Committee (46943/29.11.2021) and each participant provided written informed consent.

### 2.2. Detection of the SARS-CoV-2 IgG S Protein-Specific Antibodies 

On the first day of hospitalization, blood serum samples were collected from each patient for the semiquantitative determination of the SARS-CoV-2 IgG S protein-specific antibodies with lateral flow immunochromatographic assays (Rapid Test 2019-nCoV IgG, ProGnosis Biotech, Larissa, Greece).

5 μL of serum per sample was injected into a test tube containing dilution buffer. A strip was then immersed in the tube for 15 min. Subsequently, the strips were scanned in the S-flow reader to interpret the results. The scanner could automatically calculate the ratio (T/C) by measuring the density of the test (T) and control (C) lines of the strip. Eight standards of recombinant antibodies were used in order to create the standard/ratio curve for the anti-S Ig semiquantification. A strip in which no colored line appeared in the control band was considered invalid.

In terms of diagnostic specificity, 468 samples of pre-pandemic COVID-19 patients were analyzed, with 100% specificity. A study was conducted with 122 patients who had clinical symptoms of COVID-19 and a positive PCR result for diagnostic sensitivity. The sensitivity was calculated to be 96.72% (Rapid Test 2019-nCoV IgG, V1430, Version 24 September 2021/rev.01, ProGnosis Biotech, Larissa, Greece).

Additional blood and serum samples were collected upon hospital admission for the evaluation of the following hematological and biochemical parameters: white blood cells (WBC), lymphocytes, platelets (PLT), C-reactive protein (CRP), creatinine, urea, aspartate transaminase (SGOT), alanine transaminase (SGPT), lactate dehydrogenase (LDH), ferritin, and creatine kinase (CPK).

### 2.3. Statistical Analysis

The SPSS v 19.0 software (IBM) was used to conduct the statistical analysis. Data distribution was assessed using the Kolmogorov–Smirnov normality test. The independent samples *T*-Test and the Mann–Whitney test were used to determine significant differences of parametric and non-parametric data, respectively, between two groups. Associations between categorical variables were determined with the Fisher’s Exact Test. Correlations between quantitative variables were measured with the Pearson (r) or the Spearman (ρ) coefficients as appropriate. Logistic regression was used for the analysis of multiple variables influencing the presence of anti-S SARS-CoV-2 antibodies upon admission and the outcome of COVID-19 disease. All the variables with significant univariate associations were entered into the analysis in a single step (method selection: Enter). Statistical significance was set at the *p* < 0.05 level.

## 3. Results

### 3.1. Baseline Characteristics of the Study Population

The mean age of participants was 72.44 ± 1.22 years. Seventy-three subjects had received the BNT162b2/Pfizer vaccine, 22 the Vaxzevria, ChAdOx1-S/AstraZeneca vaccine, and 3 the Johnson & Johnson’s Janssen COVID-19 Vaccine (information regarding the type of COVID-19 vaccine was unavailable for four subjects). The mean number of days since completion of vaccination was 159.03 ± 6.35. The mean real time PCR cycle threshold (Ct) value was 20.01 ± 0.54. The baseline laboratory characteristics of the study population are presented in Table 2.

### 3.2. Factors Influencing SARS-CoV-2 IgG S Antibody Responses

Twenty-seven subjects had a negative antibody test upon hospital admission. In the remaining patients, the anti-S IgG antibodies ranged from 0.09AU to >12.48AU. A strong positive correlation was observed between the SARS-CoV-2 IgG S levels (when detectable) and Ct values upon admission (ρ = 0.592, *p* < 0.001). Compared to cases with detectable antibody levels, cases with a negative antibody test were older (*p* = 0.039) and had a higher creatinine level on admission (*p* = 0.001). The same group of patients was also observed to have lower Ct values (*p* < 0.001) and a shorter duration between symptom onset and hospital admission (*p* < 0.001). The absence of anti-S SARS CoV-2-antibodies on the first day of hospitalization was also associated with the presence of diabetes (*p* = 0.006), PaO2/FiO2 (PF) ratio values <150 mm Hg (*p* = 0.014), and death (*p* = 0.019) (Table 2). The Ct values and time between symptom onset and hospitalization remained significant in the multiple regression analysis (*p* = 0.023 and *p* = 0.025, respectively) (Table 3).

### 3.3. Factors Influencing the Outcome of COVID-19 Disease in Fully Vaccinated, Hospitalized Patients

Out of the 102 patients admitted to the hospital, 88 were discharged and 14 died. All deceased subjects had received SARS-CoV-2 mRNA vaccines (*p* = 0.035). Poor disease outcome was associated with older age (*p* = 0.003), lower Ct values (*p* = 0.036), a shorter duration between symptom onset and hospital admission (*p* = 0.007), and lower BMI (*p* = 0.029). Non-deceased patients were more likely to have hypertension (*p* = 0.014) and PF ratio values > 150 mm Hg (*p* < 0.001) (Table 2). The PF ratio was identified by the multiple logistic regression model as an independent predictor of in-hospital mortality (*p* = 0.001) (Table 4). The “vaccine type” variable was not included in the multiple regression analysis since none of the deceased patients had received a viral vector COVID-19 vaccine.

## 4. Discussion

To our knowledge, this is the first study to assess several factors affecting SARS-CoV-2 IgG S antibody responses in fully vaccinated COVID-19 patients needing hospitalization due to severe COVID-19 disease. We found that older age, lower Ct values, and a shorter duration between symptom onset and hospital admission were associated with a lack of anti-S SARS-CoV-2 antibodies and poor clinical outcomes of COVID-19 disease.

The available evidence suggests that humoral and cellular immune responses are impaired in aged individuals, resulting in decreased vaccine responses [15]. Age has been reported to be inversely correlated with neutralizing antibody responses following the first immunization dose of BNT162b2, a finding that was particularly evident for individuals over 80 years [16]. The investigation of humoral immunity after two doses of BNT162b2 and mRNA-1273 vaccines has indicated that adults aged 18–55 years are more responsive to vaccination and maintain humoral immunity longer compared to individuals who are older than 70 years [17]. In addition, the anti-S SARS-CoV-2 immunoglobulin G antibody titers were found to be significantly lower in elderly vaccinees over the age of 80 years, with 31.3% of them having no detectable neutralizing antibodies after the second vaccine dose [18]. These observations and our findings underline the need for prioritizing booster COVID-19 vaccination in the elderly population.

Regarding the SARS-CoV-2 viral load, it has been shown that fully vaccinated subjects with breakthrough infections have a comparable peak viral load to those who are unvaccinated [19]. However, peak viral load increased with age, highlighting the importance of adjusting for age when comparing the two groups [20]. In our cohort of fully vaccinated inpatients, lower Ct values, which are indicative of higher viral loads, were associated with the absence of anti-S SARS-CoV-2 antibodies upon admission, both in the univariate and multiple regression analysis. The shorter number of days between symptom onset and hospital admission could account for the lower Ct values in the group of cases with a negative antibody test whose disease progressed faster, requiring earlier hospitalization. Lower Ct values were also observed in the group of deceased subjects, yet this finding did not remain significant in the multiple regression analysis. With respect to the positive correlation between anti-S SARS-CoV-2 IgG levels and Ct values upon admission, it has been reported that higher Ct values following BNT162b2 vaccination are associated with higher IgG concentrations [21].

The PF ratio was identified as an independent predictive variable of mortality in our cohort of fully vaccinated COVID-19 inpatients. Both the PF ratio and the ratio between standard PaO2 over FiO2 (STP/F) have been described as accurate predictors of acute respiratory failure outcome in COVID-19 patients [22].

Despite the fact that COVID-19 is characterized by atypical pneumonia followed by severe respiratory failure, about 10% of COVID-19 inpatients have been reported to endure acute kidney injury, which is linked to a poor prognosis [23]. It has been reported that changes in serum creatinine during the early stage of admission could predict mortality during hospitalization in COVID-19 patients [23,24]. In our study, serum creatinine levels upon admission were not predictive of in-hospital mortality, but subjects with a negative anti-S SARS-CoV-2 antibody test had higher creatinine levels on the first day of hospitalization compared to participants with detectable antibody levels, albeit not independently from other factors. Of interest, a multicenter cohort study of 543 subjects on hemodialysis and 75 healthy subjects found that both the humoral and cellular immune responses to SARS-CoV-2 vaccination were significantly impaired in the patients’ group [25].

Findings with respect to diabetes were recently published as sub-study results for 92 patients of the CoVax study [26]. Diabetes mellitus, particularly T2D, is a prevalent comorbidity that considerably increases the risk of mortality in COVID-19 patients [27]. The immune system is thought to cause transitory alterations in systemic metabolism as a defense against viral infection. This mechanism is impaired in subjects with T2D, reducing the antiviral immune response [28].

Comorbidities related to a metabolic syndrome such as T2D, obesity, and hypertension are also characterized by low-grade chronic inflammation, which leads to immune system dysregulation and increased susceptibility to severe COVID-19 disease [3]. Paradoxically, in our cohort, deceased patients were less likely to have hypertension and their mean BMI was lower compared to non-deceased participants. The “obesity paradox” has been described in patient cohorts with several diseases including, but not limited to, T2D, hypertension, and chronic kidney disease [29]. However, caution is needed in interpreting these data given that all possible confounding variables should be taken into account and measured prospectively [29].

The remaining factors investigated in our study were not predictive of either the SARS-CoV-2 IgG S antibody responses or the outcome of COVID-19 disease in fully vaccinated inpatients. Gender and sex-specific effects have been reported to induce different immunization and adverse events outcomes [4,30]. The recent implementation of a within-host mathematical model of vaccine dynamics from lipid nanoparticle-formulated COVID-19 mRNA vaccines found no difference between sexes in the long-term duration of humoral immunity [17]. Regarding the “place of residence” variable, it has been reported that individuals living in highly deprived areas have increased odds of post-vaccination SARS-CoV-2 infection following the first vaccine dose [31].

It would be of great importance to ensure that the positive antibody test is a resultant of immunity induced exclusively by SARS-CoV-2 vaccination. Anti-S SARS-CoV-2 antibodies are produced in response to vaccine administration and/or COVID-19 infection. Thus, our method could not distinguish between post-vaccine response and infection. We also acknowledge that this is a single center study with a relatively small sample. Future studies should evaluate the parameters that have an impact on the vaccine-induced immunity against SARS-CoV-2 in subjects with breakthrough infections not requiring hospitalization.

## 5. Conclusions

Host- (age) and disease-associated factors (Ct values, time between symptom onset and hospitalization, and PF ratio) can predict SARS-CoV-2 IgG S responses and clinical outcomes in hospitalized COVID-19 patients with breakthrough SARS-CoV-2 infection post vaccination.

## Figures and Tables

**Table 1:** The factors that were investigated in the present study in terms of their effect on SARS-CoV-2 IgG S antibody responses.

**General Information**	**Lifestyle**	**Comorbidities**
Age Sex BMI Number of family members Days with symptoms from onset until admission Occupation	Smoking (pack/years) Alcohol (weekly consumption) Exercise (40 min/week) Fruit and vegetable consumption (Yes/No)	Diabetes (Yes/No) Coronary artery disease (Yes/No) Arterial hypertension (Yes/No) COPD-asthma (Yes/No) Obstructive apnea syndrome (Yes/No) Renal disease (Yes/No) Neoplasia (Yes/No) Autoimmune disease (Yes/No)
**Drug Consumption**	**Vaccine Information**	**Antibodies**
Immunosuppressive drugs before vaccination (Yes/No) probiotics (Yes/No) Antibiotics taken one week before or after vaccination (Yes/No) Nonsteroidal anti-inflammatory drugs one week before or after vaccination (Yes/No) Vitamin intake one week before or after vaccination (Yes/No)	Anxiety about vaccination (Yes/No) Vaccine type Vaccine doses Day since last dose Symptoms after vaccination (fever > 38, hand pain, arthralgia/myalgia)	Presence of anti-S SARS-CoV-2 antibodies on admission (Yes/No)

**Table 2:** Baseline characteristics of the study population (*N* = 102).

	Cases With A Negative Antibody Test (*N* = 27)	Cases with Detectable Antibody Levels (*N* = 75)	*p* Value	Deceased Patients (*N* = 14)	Non-Deceased Patients (*N* = 88)	*p* Value
Age, years, median ± SD *	75 ± 11.1	73 ± 12.4	0.039	82 ± 8.3	71 ± 12.2	0.003
Body mass index, median ± SD	26 ± 4.5	26.9 ± 4.1	ns	24.8 ± 3.1	27.2 ± 4.1	0.029
Male sex (%)	18	52	ns	11.9	57.4	ns
Residence, urban (ratio)	0.7	0.76	ns	0.85	0.74	ns
Use of probiotics (ratio)	0	0.03	ns	0	0.02	ns
Vitamin use (ratio)	0.26	0.16	ns	0.31	0.16	ns
Weekly exercise (ratio)	0.5	0.72	ns	0.61	0.68	ns
PaO2/FiO2 (PF) ratio < 150 mm Hg (ratio)	0.48	0.22	0.014	0.93	0.2	<0.001
Corticosteroids use before vaccination (ratio)	0.08	0.03	ns	0.07	0.04	ns
COVID-19 mRNA vaccination (ratio)	0.76	0.74	ns	1	0.70	0.035
Vaccination anxiety (ratio)	0.13	0.16	ns	0.23	0.15	ns
Symptoms post vaccination (ratio)	0.22	0.41	ns	0.15	0.39	ns
Days since last vaccination dose, median ± SD	168 ± 63.1	163 ± 59.6	ns	181 ± 77.1	163 ± 58.5	ns
Days from symptom onset to admission, median ± SD	4 ± 2.3	6 ± 2.3	<0.001	5 ± 2.2	6 ± 2.45	0.007
Hospitalization days, median ± SD	6 ± 9.2	6 ± 5.2	ns	6 ± 7.8	6 ± 6.3	ns
Laboratory testing						
SARS-CoV-2 Cycle threshold, median ± SD	15.56 ± 4.15	19.84 ± 5.5	<0.001	16.26 ± 4.2	19.25 ± 5.57	0.036
Detection of anti-S SARS-CoV-2 IgG responses (ratio)	-	1	-	0.43	0.77	0.019
anti-S SARS-CoV-2 IgG titers (A.U.), median ± SD	-	2.83 ± 3.57	-	0 ± 1.53	1.49 ± 3.6	0.001 **
White blood cells (×10^9^/L), median ± SD	6100.00 ± 3758.48	7600.00 ± 3734.09	ns	7950.00 ± 3877.3	7000.00 ± 3768.4	ns
Lymphocytes (×10^9^/L), median ± SD	640.00 ± 340.70	740.00 ± 1312.97	ns	595.00 ± 330.6	720.00 ± 1215.1	ns
Platelets (×10^9^/L), median ± SD	205000.00 ± 58002.23	219000.00 ± 83154.72	ns	201000.00 ± 85309.13	215000.00 ± 77491.78	ns
C-Reactive protein (mg/dL), median ± SD	4.72 ± 10.67	8.31 ± 6.60	ns	13.18 ± 12.9	6.63 ± 6.45	ns
Creatinine (mg/dL), median ± SD	1.19 ± 0.92	0.9 ± 0.38	0.001 **	0.97 ± 1.27	0.94 ± 0.37	ns
Urea (mg/dL), median ± SD	46.50 ± 41.80	38.00 ± 38.96	ns	44.2 ± 55.2	38.2 ± 36.27	ns
Serum glutamic-oxaloacetic transaminase (IU/L), median ± SD	27.70 ± 34.80	28.00 ± 26.10	ns	34.7 ± 31.9	27.9 ± 27.5	ns
Serum glutamic pyruvic transaminase (IU/L), median ± SD	22.00 ± 27.73	24.00 ± 30.69	ns	21.65 ± 24.7	24.1 ± 27.2	ns
Lactate Dehydrogenase (IU/L), median ± SD	314.00 ± 221.34	357.00 ± 154.93	ns	371.5 ± 275.77	335.00 ± 143.81	ns
Ferritin (ng/mL), median ± SD	619.70 ± 615.67	588.20 ± 722.72	ns	653.50 ± 1189.80	581.75 ± 546.09	ns
Creatine Kinase (U/L), median ± SD	99.00 ± 637.14	97.00 ± 133.20	ns	115.50 ± 768.43	94.50 ± 212.89	ns
Comorbidities						
Diabetes (ratio)	0.44	0.15	0.006	0.38	0.22	ns
Coronary disease (ratio)	0.3	0.2	ns	0.3	0.2	ns
Hypertension (ratio)	0.48	0.5	ns	0.16	0.55	0.014
Asthma, Chronic obstructive pulmonary disease (ratio)	0.12	0.10	ns	0.15	0.1	ns
Obstructive Sleep Apnea Syndrome (ratio)	0	0.01	ns	0.08	0	ns
Renal disease (ratio)	0.08	0.03	ns	0	0.05	ns
Cancer (ratio)	0.08	0.07	ns	0.07	0.07	ns
Autoimmune disease (ratio)	0.13	0.07	ns	0.08	0.1	ns

* SD; Standard deviation, ** Mann-Whitney U Test.

**Table 3:** Results of the multiple regression analysis with respect to the variables affecting the presence of anti-S SARS-CoV-2 antibodies upon admission.

Variables in the Model	B	S.E.	Wald	df	*p* Value	Exp (B)	95% C.I. for EXP(B) **	
							Lower	Upper
age	0.006	0.027	0.047	1	0.828	1.006	0.954	1.061
Ct	0.249	0.110	5.139	1	0.023	1.283	1.034	1.591
PF atio (1)	0.758	0.668	1.288	1	0.256	2.134	0.576	7.899
Days WSBH *	0.387	0.173	4.990	1	0.025	1.472	1.049	2.067
CREATININE	−0.736	0.588	1.569	1	0.210	0.479	0.151	1.515
Diabetes (1)	0.598	0.694	0.745	1	0.388	1.819	0.467	7.083
Constant	−6.044	3.266	3.425	1	0.064	0.002		

Dependent variable: detection of anti-S SARS-CoV-2 antibodies upon admission; Parameter coding (1): non-diabetic; PF ratio > 150 mm Hg. * Days between symptom onset and hospitalization, ** B; the coefficient for the constant, S.E.; the standard error for B, Wald; the Wald chi-square test, df; the degrees of freedom for the Wald chi-square test, Exp(B); The exponentiation of the B coefficient, C.I; confidence interval.

**Table 4:** Results of the multiple regression analysis with respect to the variables affecting the outcome of COVID-19 disease in fully vaccinated, hospitalized patients.

Variables in the Model	B	S.E.	Wald	df	*p* Value	Exp (B)	95% C.I. for EXP(B) **	
							Lower	Upper
age	0.069	0.053	1.721	1	0.190	1.071	0.967	1.188
Ct	−0.216	0.148	2.135	1	0.144	0.806	0.603	1.077
Days_WSBH *	−0.059	0.306	0.037	1	0.847	0.943	0.517	1.718
BMI	−0.235	0.160	2.159	1	0.142	0.791	0.578	1.081
antibodies (1)	−0.275	1.194	0.053	1	0.818	0.760	0.073	7.893
PF_Ratio (1)	−4.156	1.442	8.305	1	0.004	0.016	0.001	0.265
Hypertension (1)	1.115	1.185	0.885	1	0.347	3.050	0.299	31.135
Constant	4.095	8.290	0.244	1	0.621	60.068		

Dependent variable: Mortality; Parameter coding (1): No detection of antibodies, non-hypertensive, PF ratio > 150 mm Hg. * Days between symptom onset and hospitalization, ** B; the coefficient for the constant, S.E.; the standard error for B, Wald; the Wald chi-square test, df; the degrees of freedom for the Wald chi-square test, Exp(B); The exponentiation of the B coefficient, C.I; confidence interval.

## Data Availability

The data that support the findings of this study are available upon request from the corresponding author.
